# Cortical Organoids to Model Microcephaly

**DOI:** 10.3390/cells11142135

**Published:** 2022-07-07

**Authors:** Sarah Farcy, Alexandra Albert, Pierre Gressens, Alexandre D. Baffet, Vincent El Ghouzzi

**Affiliations:** 1 Institut Curie, PSL Research University, CNRS UMR144, F-75005 Paris, France; sarah.farcy@curie.fr; 2 NeuroDiderot, Inserm, Université Paris Cité, F-75019 Paris, France; alexandra.albert@inserm.fr (A.A.); pierre.gressens@inserm.fr (P.G.)

**Keywords:** primary microcephaly, post-natal microcephaly, brain organoids, neocortex development, induced pluripotent stem cells (iPSCs), Golgipathies

## Abstract

How the brain develops and achieves its final size is a fascinating issue that questions cortical evolution across species and man’s place in the animal kingdom. Although animal models have so far been highly valuable in understanding the key steps of cortical development, many human specificities call for appropriate models. In particular, microcephaly, a neurodevelopmental disorder that is characterized by a smaller head circumference has been challenging to model in mice, which often do not fully recapitulate the human phenotype. The relatively recent development of brain organoid technology from induced pluripotent stem cells (iPSCs) now makes it possible to model human microcephaly, both due to genetic and environmental origins, and to generate developing cortical tissue from the patients themselves. These 3D tissues rely on iPSCs differentiation into cortical progenitors that self-organize into neuroepithelial rosettes mimicking the earliest stages of human neurogenesis in vitro. Over the last ten years, numerous protocols have been developed to control the identity of the induced brain areas, the reproducibility of the experiments and the longevity of the cultures, allowing analysis of the later stages. In this review, we describe the different approaches that instruct human iPSCs to form cortical organoids, summarize the different microcephalic conditions that have so far been modeled by organoids, and discuss the relevance of this model to decipher the cellular and molecular mechanisms of primary and secondary microcephalies.

## 1. Introduction

How the human brain develops from the embryo to adulthood, and to what extent its complexity reflects the specificity of the cellular and molecular mechanisms that are selected through evolution are fascinating scientific issues. In this respect, several species-specific characteristics could explain the large expansion of the human neocortex. During its development, several types of progenitors gradually emerge and are distinguished by their morphology and their position in the different germinal zones [1,2]. The ventricular zone (VZ), closest to the lumen of the neural tube, contains the apical radial glial cells (aRGs), a progenitor type with epithelial features and present in all mammals. The subventricular zone (SVZ), located basally to the VZ, hosts two progenitor cell types, collectively referred to as basal progenitors (BPs). While intermediate progenitors (IP) are a group of transient amplifying BPs that are common to humans and rodents, the SVZ in humans contains another, highly abundant subpopulation of BPs that are very rare in mice—the basal radial glial cells (bRG). bRG cells share many characteristics with aRG cells but are delaminated from the neuroepithelium. As they are highly proliferative, bRG cells are thought to be of critical importance for brain size expansion, especially in gyrencephalic species [3]. Underlying the differences in progenitor abundance and proliferative capacity in gyrencephalic species, many specific gene expression profiles and cell cycle regulation features have been reported [4,5,6].

Organoids refer to 3D structures that are grown in vitro from stem cells and made up of several organ-specific cell types that are capable of self-organizing in a coordinated manner and retaining certain functionalities of the organ [7]. In this context, the ability of human pluripotent stem cells (PSC) to form neuroepithelial tissue and to self-organize into structures mimicking the apico-basal polarity of the neocortex (and to some extent, retaining the spatial-temporal memory of the sequential steps of neurogenesis) has been determinant in the current rise of human organoids as a model of brain development [8,9,10].

At the same time, the increasing expansion of the human-induced pluripotent stem cell (iPSC) technology has made it possible to generate organoids from patients carrying genetic mutations, with immense prospects for all developmental diseases. Among the many neurodevelopmental pathologies that have been studied using brain organoid models, hereditary microcephaly, defined as a brain growth deficit of genetic origin, appears to be the ideal pathological paradigm for understanding the cellular and molecular mechanisms of human brain development [11].

The full process of human brain development—from the onset of neurogenesis to the completion of brain maturation—is extremely long, as it extends from early embryogenesis to adulthood. Thus, primary microcephaly, which by definition is congenital, reflects brain defects occurring during the embryo-fetal period, while secondary microcephaly, which is postnatal, usually results from maturation deficits. In both situations, organoids turn out to be promising models, because in addition to faithfully mimicking the early stages of development, the latest technological advances are now beginning to enable the study of later stages such as synaptogenesis, functional outputs of post-mitotic neurons, and connectivity between various brain areas.

Here, we review the various ways to instruct pluripotent stem cells to form cortical organoids and discuss what organoid modeling has offered so far to the understanding of the cellular and molecular mechanisms of primary microcephaly. We also discuss the current challenges and technical advances allowing the study of the long-term maturation of cortical organoids and their relevance in modeling secondary microcephaly.

## 2. Generation of Cerebral Organoids

Cerebral organoids are PSC-derived 3-dimentional tissues, cultivated in vitro, that mimic the development of the brain. They differ from neurospheres in that they are not simple free-floating induced aggregates, but more complex structures that are organized with an apico-basal polarity and specific cellular zones [12]. The Sasai group was the first to anticipate and achieve the differentiation of dorsal telencephalic precursors from stem cell aggregates, called embryoid body (EB)-like aggregates [13], and to show that they can spontaneously self-organize into apico-basally polarized cortical tissue [14]. Since then, all protocols for cerebral organoid generation largely rely on their self-organization properties, though pre-patterning steps with specific molecules can be performed, depending on the chosen method. Unguided methods were established by Lancaster and Knoblich [15], who embedded EBs into Matrigel, an ECM-based scaffold that allows tissue growth in 3D. EBs autonomously acquire an ectodermal (and progressively neuro-ectodermal) fate, leading to the formation of large neuroepithelial units surrounding lumens. Another key development was the culture of the growing organoids under agitation, which can be achieved using either spinning bioreactors or orbital shakers, allowing improved oxygenation of the sample, and leading to the formation of much larger ventricle-like structures [16]. Finally, the addition of dissolved Matrigel into the culture media of organoids to reconstitute the basement membrane substantially improved the quality of organoid generation, leading in particular to the formation of a well-defined cortical plate (CP) [17].

Because of the absence of pre-patterning, unguided methods lead to the unpredictable formation of a variety of cerebral regions, including dorsal and ventral forebrain, midbrain, choroid plexus, or retinal tissue. Guided methods for cerebral organoid generation consist of applying specific patterning agents at the initial stages of the protocol, in order to obtain uniform differentiation into specific brain regions [18]. To generate forebrain organoids, a reliable method consists of inhibiting SMAD signaling using BMP inhibitors, which block mesodermal and endodermal differentiation [19,20,21]. This treatment can be applied from day 0, especially if EBs are generated from entire colonies, rather than from single cell suspensions. The addition of WNT inhibitors to further repress mesoderm and promote anterior ectoderm is also commonly used [22]. A variety of guided protocols based on SMAD pathway inhibition have been reported, with different characteristics. The Ming and Song group devised a protocol relying on a miniaturized spinning bioreactor for the generation of dorsal forebrain organoids, as well as other cerebral regions [20]. These 3D-printed reactors allow for culture into stackable 12-well plates, which increases the number of conditions that can be tested, while reducing costs and space requirements [23]. Another original feature of this method is the use of Matrigel “cookies” instead of individual drops, in which large numbers of EBs can be grown. The main advantage of this approach is that it facilitates the embedding and retrieval of organoids from Matrigel, reducing workload.

Interestingly, several protocols do not rely on Matrigel, at least at the initial steps [24]. Rather, well-crafted culture media appear sufficient to initiate 3D growth and differentiation of these free-floating cultures [25,26]. An advantage of such an approach is that it is not dependent on the inherent variability of Matrigel, whose composition slightly varies from batch to batch. In the widely used protocol from the Pașca lab, forebrain organoids are grown in an ultra-low attachment plate and cultured in suspension without Matrigel or any form of agitation [21,26]. Organoids are furthermore exposed to the EGF and FGF growth factors to stimulate their proliferation and growth up until day 25.

To generate pure dorsal forebrain (cortical) organoids, the samples can be treated at this stage with an SHH inhibitor (CycA) [27]. A variety of other protocols have been developed to generate different brain regions, including midbrain, hypothalamus, cerebellum, hippocampus, or choroid plexus [15,16]. While these methods enable the improvement of reproducibility, they do not allow an investigation of the interaction between brain regions. A major step forward towards this goal was the development of fused ventral and dorsal forebrain organoids, so-called assembloids, enabling the modeling of processes such as ventral-to-dorsal interneuron migration [27,28,29].

Overall, these methods enable mimicking of the early stages of human neurogenesis with a surprising level of similarity. In the dorsal forebrain, the presence of many cell types has been confirmed using single-cell RNAseq methods, including apical and basal radial glial (aRG and bRG), intermediate progenitors, several subtypes of pyramidal neurons, as well as glial cells after three months of culture [25]. These cells appear to follow a good organization pattern, with aRG forming a dense neuroepithelium apically, and bRG cells being scattered basally. Neurons undergo migration, form rudimentary layers and begin to establish neural circuits. Therefore, cerebral organoids can be used to model human neurological diseases, especially developmental ones such as microcephaly.

## 3. Modeling Primary Microcephaly Using Cerebral Organoids

The use of cerebral organoids to model human microcephaly responds to two major limitations of animal models. First, they allow the study of human-specific features that may not be recapitulated in mouse. In support of this, several mouse models for human microcephaly have failed to fully recapitulate the phenotype [30,31,32,33]. One of the most obvious differences between human and mouse is the abundance of bRG cells in human fetal tissue that account for the majority of supragranular layer neuron production [34]. Moreover, the rare bRG cells that are present in mouse may not have the same proliferative capacity in humans [35,36]. It is nevertheless important to keep in mind that the abundance of bRG cells remains limited in organoids as compared to human fetal tissue, though newly developed protocols improve this aspect, including the sliced neocortical organoids (SNOs) method (detailed in chapter 5.3) [37]. A second major advantage of cerebral organoids is that they enable the modeling of polygenic diseases, or diseases without known causative mutation, which are difficult to model in mouse [38,39]. Unlike animal models, cerebral organoids are furthermore highly amenable to physico-chemical manipulations, allowing testing, in a controlled set-up, of the effect of various molecules on their development. For all these reasons, they allow a reduction in the number of animals that are used in research, an increasingly critical directive that is given by funding bodies.

A number of pioneer studies have begun to demonstrate the potential of cerebral organoids in modeling human microcephaly (Supplementary Table S1). The first proof of principle that cerebral organoids can be used to model microcephaly came from the original Lancaster and Knoblich study [15]. The authors reprogrammed patient cells carrying mutations in *CDK5RAP2* into iPSCs and generated organoids. CDK5RAP2 is a centrosomal protein that is required for pericentriolar material recruitment and mitotic spindle assembly and, when mutated, is a frequent cause of microcephaly [40]. Mutant cerebral organoids displayed smaller neuroepithelial regions and contained less progenitor cells and more neurons, suggesting premature differentiation. The authors observed more oblique divisions of the aRG cells, which would lead to their delamination. While this phenomenon is a natural event in the human brain that generates bRG cells [41], its early occurrence would lead to an impaired tangential expansion of the neuroepithelium. A recent report in mouse demonstrated that early delaminating cells can re-integrate into the neuroepithelium [42]. Whether the same occurs in human is unknown, but this result may point to other defects participating in the microcephaly phenotype. For instance, mitotic spindle organization defects could lead to abnormal chromosome segregation and apoptosis.

Mutations in the *ASPM* gene (abnormal spindle-like microcephaly-associated) are the most common cause of primary microcephaly in humans [43,44,45]. Again, mouse models for *ASPM* loss of function poorly recapitulated the human phenotype [33], though a recent study in ferrets demonstrated closer defects, particularly regarding cortical surface area [46]. As a small gyrencephalic animal, it is worth noting that the ferret is an interesting model with which to study microcephaly, especially as it contains a similar proportion and morphotypes of bRG cells than those of humans [47]. However, available transgenic ferret lines are still very limited. ASPM patient-derived cerebral organoids were also shown to display severe growth defects [48]. The organoids were extremely disorganized with barely recognizable neuroepithelial regions in which proliferation rates were very low. Very few PAX6+ (RG cells) progenitors and CTIP2+ (upper layer) or SATB2+ (deep layer) neurons were present, raising the question of the differentiation status of these mutant organoids. More work is required to identify the molecular consequences of *ASPM* mutation, in particular in human bRG cells [23,26,49].

Mutations in the gene coding for centrosomal-associated-P4.1 protein (CPAP) cause Seckel syndrome: primary microcephaly that is coupled with a reduction in body size. Using a combination of patient fibroblasts, iPS-derived neural stem cells (NSCs) and cerebral organoids, Gabriel and colleagues identified a role for CPAP in primary cilia disassembly and control of the progenitor cell pool [50]. Patient-derived organoids displayed smaller neuroepithelial regions with disorganized ventricular zones and, notably, larger lumen. No difference in apoptotic cell death was observed, but the organoids appeared to undergo premature differentiation, with reduced PAX6+ progenitors and increased TUJ1+ neurons. Mutant progenitors had longer cilia and experiments in fibroblasts and NSCs showed delayed cilia disassembly. Mechanistically, CPAP was shown to act as a scaffold to recruit OFD1, Nde1 and Aurora A, members of the machinery for cilia disassembly. Mutated CPAP did not localize to the cilia, therefore impairing its disassembly. In agreement with the ciliary defects, the authors furthermore identified defective cell cycle progression and premature differentiation. Based on these observations, they proposed that, upon *CPAP* mutation, impaired cilia disassembly leads to the reduced proliferation of progenitor cells and, as a consequence, to their premature cell cycle exit and differentiation [51,52]. While cerebral organoid models cannot address the complexity of multi-organ defects that are associated with syndromes such as Seckel, investigating the brain-specific aspects of such pathologies remains highly relevant and powerful. As highlighted in this study, they allow identification of the molecular alterations causing reduced brain growth. Such work provides powerful insight to identify the causes of the alterations that are present in other organs.

A role for cilia disassembly was further demonstrated through the investigation of the WDR62-CEP170-KIF2A pathway [53]. Using CRISPR/Cas9 to generate loss of function iPS lines, the authors demonstrated the altered growth of WDR62 KO cerebral organoids. As is consistently reported for smaller organoids, the ventricular zone was thinner, with less SOX2+ and PAX6+ cells. These cells proliferated less, and this was accompanied by increased differentiation. No increase in apoptosis was detected. Notably, WDR62 loss of function also caused a decrease in cycling bRG cells. WDR62 was reported to interact with the centrosomal protein CEP170 and to recruit it to the basal body of the primary cilia [53]. Both factors were shown to participate in cilia disassembly, via basal body recruitment of the microtubule depolymerizing factor KIF2A. Strikingly, KIF2A overexpression rescued, or partially rescued, cilia length, cell cycle progression, premature differentiation and even organoid size in WDR62 KO background.

An entirely different molecular cause of microcephaly was recently highlighted through the study of the microcephaly-associated factor NARS1, a Class IIa tRNA synthetase [54]. The authors identified novel patients with biallelic variants in *NARS1*, either homozygous (consanguineous families) or compound heterozygous (non-consanguineous). These patients displayed severe microcephaly, cerebellar atrophy and ventriculomegaly. Mutations affected NARS1 stability, leading to global protein synthesis defects. Patient-derived organoids were generated following a protocol that was modified from the Sasai and Pașca labs (Matrigel-independent at induction stage and without agitation). The growth of these organoids was severely affected, with impaired formation of the neuroepithelial structures (rosettes). *NARS1* mutations led to the reduced proliferation of neuronal progenitors, leading to reduced neuronal production. Moreover, increased levels of apoptosis were also reported. Surprisingly, single cell RNAseq data pointed to an altered cell fate towards astrocytes at the expense of neurons in mutant organoids. The mechanisms linking protein synthesis defects to proliferation and fate defects remains to be addressed.

**Figure 1:**
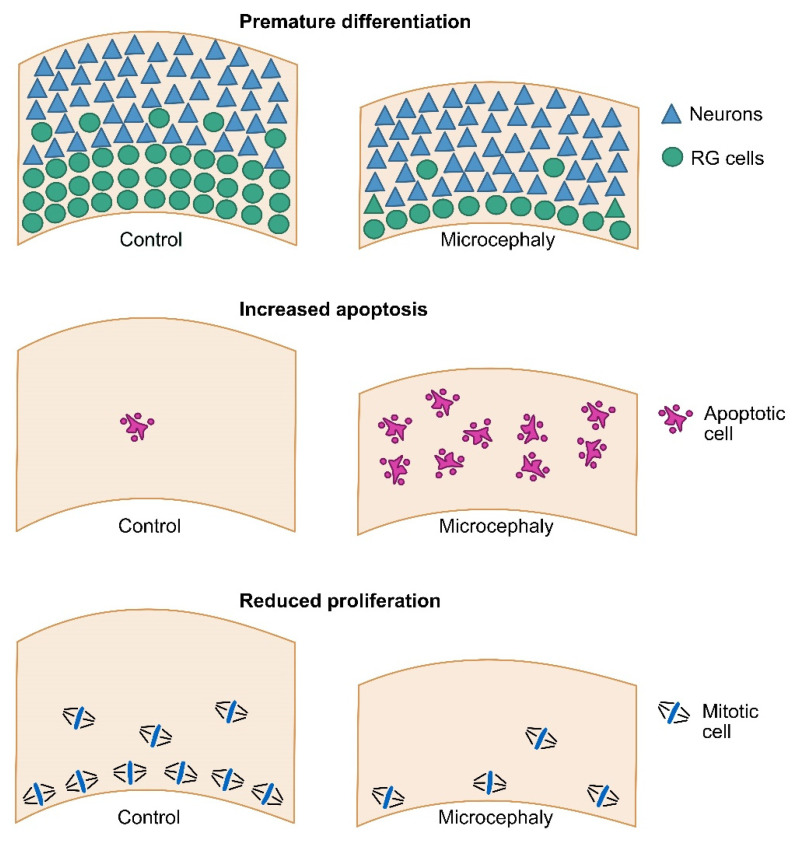
Major cellular causes of primary microcephaly. Three major causes of primary microcephaly have been reported, leading to a reduced total number of cells at birth. Premature differentiation refers to an excessive early generation of neurons, leading to an exhaustion of the progenitor pool. This phenomenon can lead to a transient excess of neurons, but ultimately causes reduced neuronal production. Increased apoptotic cell death can occur in the newborn neurons or in the progenitor cells. In the former case, neuronal production is not affected, but eventually dies. In the latter case, neuronal production is directly affected. Reduced proliferation refers to an increased duration of the cell cycle, or to an exit from the cell cycle. This typically leads to a reduced mitotic index, which can be observed in apical or basal progenitors. A slower cell cycle directly reduces the number of neurogenic cell divisions. Interestingly, a slower cell cycle can also trigger premature neuronal differentiation, and therefore progenitor depletion.

While all these examples demonstrated the power of cerebral organoids to model microcephaly with genetic causes, another promising avenue is the modeling of microcephaly due to environmental causes. In particular, several studies have taken advantage of cerebral organoids to investigate the specific tropism of the Zika virus (ZIKV) for the developing neocortex and how it affects human neurogenesis [55]. ZIKV has recently been responsible for an outbreak of microcephaly, especially in South America [56]. Strikingly, infection of cerebral organoids with ZIKV revealed a specific infection of RG cells, especially within the ventricular zone [57,58,59]. This resulted in altered cell cycle progression, as well as strong apoptotic cell death, leading to the altered growth of organoids. Overall, works using cerebral organoids, together with mouse models, have identified three major cellular causes of primary microcephaly: premature neuronal differentiation, increased apoptosis and reduced progenitor proliferation (Figure 1). As described above, the underlying molecular causes can be very diverse, and are the subject of current intense investigation.

## 4. Modeling Secondary Microcephaly Using Cerebral Organoids

Secondary microcephaly, which by definition is postnatal, results from the alteration of later and more diverse mechanisms than primary microcephaly, related to brain maturation after birth. Usually syndromic, secondary microcephaly is often only one of many clinical signs in multi-organ pathologies. As such, it is not always considered to be the most disabling feature in patients’ daily life and is thereby largely under-investigated. Nevertheless, several causes of secondary microcephalies have been modelled in cerebral organoids (Supplementary Table S2).

Rett syndrome (RTT), caused by mutations in *MeCP2*, an X-linked gene encoding a methyl-CpG binding protein that binds methylated DNA, is a severe neurological disorder affecting females. Following an apparent normal period of development, patients undergo developmental regression, with a range of neurodevelopmental defects including a deceleration of head growth in the first two years of life, associated with cognitive deterioration and seizures [60]. Modeling RTT with patient-specific forebrain organoids has revealed premature neuronal differentiation at the expense of proliferating progenitors, including a marked reduction in basal progenitors [61]. Although the stages that were studied did not exceed 12 weeks, the patients’ excitatory neurons appeared to be less mature, with altered calcium dynamics and reduced electrophysiological responses, while interneurons had a reduced capacity for migration, indicating a disequilibrium between excitation and inhibition in RTT.

Developmental and epileptic encephalopathies (DEE), a group of disorders that are characterized by intractable epileptiform activity and impaired cerebral functions, are sometimes associated with secondary microcephaly [62,63]. Using patient-derived iPSCs that were mutated in *WWOX*, a gene encoding the WW domain-containing oxidoreductase that was associated with a severe infantile epileptic encephalopathy, Steinberg et al. generated cerebral organoids aged between 10 and 24 weeks and identified impaired expression of the cortical markers TBR1, CTIP2, SATB2, and layering defects that progressively worsened at week 24 [64]. These defects were correlated with an activation of Wnt signaling. WWOX-depleted organoids further exhibited hyperexcitability, likely due to a disrupted balance between glutamatergic and GABAergic neurons.

Cohen syndrome is a rare autosomal recessive Golgipathy that is characterized by motor delays, retinal dystrophy appearing by mid-childhood, progressive severe myopia, hypotonia, joint hypermobility and secondary microcephaly that is associated with intellectual disability [65]. It results from loss of function mutations in the *VPS13B* gene, which regulates vesicle-mediated protein sorting and transport. siRNA experiments on rat primary neurons have suggested that VPS13B could be involved in neurite outgrowth through an interaction with the GTPase RAB6 [66]. More recently, recycling and internalization assays in HeLa cells have shown another role of VPS13B in transport from early endosomes to recycling endosomes via an interaction with Stx6- and Stx13-containing vesicles [67]. In a recent study by Lee et al., patient-derived iPSCs were differentiated into either forebrain-like glutamatergic excitatory neurons in 2D cultures or 3D neurospheres using dual SMAD inhibition [68]. While a transcriptomic analysis on glutamatergic neurons obtained in 2D revealed alterations of the genes involved in synaptic function, the 3D protocol did not consider stages beyond 18 days, and could thus not confirm the 2D findings. Surprisingly, neurosphere size was significantly reduced in patients from 5 days onward and the ability of neural progenitor cells to proliferate appeared affected. More work is now needed to determine at later stages whether the suggested functions of VPS13B in neuronal differentiation and synaptic function are verified in the organoid model, and whether it can be relevant for identifying pathophysiological pathways in Cohen syndrome.

The use of cerebral organoids to model human microcephaly is still in its early days and has yet to reach its full potential. In particular, focusing on molecular alterations that specifically occur in bRG cells should represent one of the most exciting added values of this model system. The recent advances in organoid protocols, particularly regarding tissue oxygenation, should enable reaching this objective. However, a number of important challenges remain.

## 5. Current Limitations and Challenges

Despite the amazing ability of iPSCs to recapitulate the diversity of neural identities and to form not only neural tissue, but spatially-temporally organized neuroepithelia that are reminiscent of in vivo development, many challenges still remain, in order to make brain organoids truly reliable and reproducible models.

### 5.1. Tissue Heterogeneity and Reproducibility Issues

Since the establishment of the first hiPSCs in 2007 [69], a high number of cell lines have been created around the world by many laboratories or biotechnology companies, and the number of scientific papers reporting the use of induced pluripotent stem cells has already largely exceeded the literature based on embryonic stem cells themselves [70]. However, the heterogeneity of the three-dimensional tissue that is generated from hiPSCs is a major concern that could compromise the reproducibility of differentiation experiments, including human cortical organoid generation. This heterogeneity occurs at several levels, from the intrinsic nature of the hiPSCs that are used for differentiation, to the various extrinsic factors, media and experimental systems that are currently used by laboratories to achieve differentiation. Numerous studies have shown that hiPSC lines retain a number of epigenetic or metabolic signatures that are characteristic of their original tissue, which could influence their differentiation potential [71,72,73]. This could explain why some cell lines are more prone to undergo neural differentiation than others. This could also explain the variable developmental outcomes in the type of neural identities that are acquired among cell lines within a given protocol. This line-dependent bias has been well illustrated in the recent study by Strano et al., who analyzed regional identity and neural cell types that were generated during cortical differentiation from a large number of independent pluripotent stem cell lines, following the same dual SMAD inhibition-based protocol [74]. Their study shows that, depending on the line that is considered, the ability to induce dorsal, ventral, rostral or caudal neuroectodermic derivatives varies greatly, and these variations appear to be sensitive to the Wnt/β-catenin pathway. Accordingly, several recent protocols of cortical induction now include modulators of this pathway in early phases of neural induction, which results in more efficient dorsalization of forebrain derivatives.

The size of the cell aggregates that are produced to form spheroids or embryoid bodies, as well as the forces that are exerted on these cells and their compactness, further influence cell–cell interactions, which can then impact differentiation trajectories [17,75]. Adding to this level of heterogeneity, various protocols have been proposed by laboratories to induce the neural differentiation of PSCs, and of hiPSCs in particular. Many extrinsic factors can influence the way that cells differentiate, such as the composition of the media, the combination of growth factors and their concentration, the timing at which they are added, and the level of oxygenation or even the relative density of the cells in the culture dish. All these parameters are under study and manipulated with the aim of generating more homogeneous and reproducible cortical differentiations. Several standardized protocols have already been developed in this sense, which, although they share common key steps and media, remain specific to each laboratory and expertise [20,25,76,77].

For all these reasons and whichever the protocol used, it appears increasingly essential to use isogenic control lines to characterize the neurodevelopmental phenotypes of a genetic mutation. The CRISPR/Cas9 editing system applied to the genome editing of hiPSCs [78] now allows the creation or correction of specific mutations that are associated with a microcephalic phenotype, thus making it possible to validate the pathogenicity of the mutation, or to provide reliable controls to analyze the associated neurodevelopmental phenotypes [79,80,81].

### 5.2. Lack or Paucity of Certain Neural and Non-Neural Derivatives

One of the major challenges of the organoid model for the study of brain development is that it may faithfully reproduce human neurogenesis at early stages of neurogenesis, but much less at late stages. The sequential appearance and the mode of division of the different progenitors, such as aRG cells, bRG cells and IP, as well as the spatial organization of the first cortical layers, is indeed relatively well respected, making the organoid model a first-rate model for the study of congenital microcephaly. In contrast, the more advanced stages of development that are valuable in understanding the mechanisms of migration, synaptogenesis and brain maturation, largely associated with postnatal microcephaly, are much more difficult to model. In particular, this is because certain derivatives are either somewhat or not represented in most of the current cortical organoid protocols. While astrocytes seem relatively well represented, several studies have shown that oligodendrocytes are very few in “traditional” organoids, whether they result from by-default or guided inductions [15,20,21,82]. Likewise, populations of interneurons, which appear in specific brain areas and then migrate to other regions such as the cortex, are under-represented in cortical organoid models. Microglial cells, which are the brain-resident macrophages acting as the immune system of the brain, or endothelial cells and pericytes, which make up the vascular network that carries and provides nutrients and oxygen throughout the whole brain, have a mesodermal origin and are also not present in current cortical models. These limitations could constitute an obstacle in using organoids to analyze brain maturation deficits which are essential to understand the postnatal outcomes of congenital microcephaly, as well as the mechanisms leading to the development of postnatal microcephaly. For example, in the FOXG1 syndrome where haploinsufficiency of the Forkhead transcription factor induces a microcephaly that is associated with severe intellectual disability, motor delay, epilepsy, and delayed myelination, both oligodendrocytes and GABAergic interneurons were found to be reduced, in addition to cortical lamination defects [83]. Similarly, a reduced activity of ATP1A3, a Na-K ATPase that is mainly expressed in cortical interneurons, has been associated with a severe developmental disability including epilepsy and postnatal microcephaly [84]. An increasing number of postnatal microcephaly appears to result from maturation defects involving defective myelination or deficient connectivity [65,85], indicating a likely contribution of oligodendrocytes and interneurons in the microcephalic phenotype. The role of microglia in shaping the developing brain (beyond its well-known immune functions), both during neurogenesis where it restricts the number of neural precursor cells [86], and at several maturation stages–such as synaptic formation [87], the phagocytosis of unnecessary synapses [88] and modulation of the neuronal network during postnatal development [89]—further underlines the need to consider these cell types in cortical organoid models.

Recent studies have begun to address these issues either by modifying existing protocols or by creating assembloids by fusing brain region-specific organoids. Madhavan et al. [90] and Marton et al. [91] have optimized a protocol from the Pașca lab [21] to generate pre-oligodendrocytes and myelinating oligodendrocytes within cortical spheroids by exposing them to PDGF, IGF-1 and T3 growth factors. Single cell RNA sequencing (scRNA-seq) and fluorescence analyses showed that the produced oligo-cortical structures contained glial clusters expressing markers of early progenitors, as well as OPCs and maturing oligodendrocytes, while preserving the global organization and intrinsic cortical patterning of organoids. These organoids were also sensitive to pro-myelinating drugs [90] or demyelinating toxic compounds [91], suggesting they could represent relevant models to study myelin-related diseases. Modeling human GABAergic interneuron migration and interaction with glutamatergic cortical neurons has also been shown to be efficient by fusing ventral and dorsal forebrain-derived structures mimicking medial ganglionic eminence (MGE) and cortical regions, respectively [27,28,29]. The resulting MGE-cortical assembloids contained interneurons forming synapses with cortical neurons, developing morphological complexity as a consequence of these interactions, and integrating into a functional microphysiological system [28]. Likewise, several methodologies were adapted to obtain microglial cells within cortical organoids. Ormel et al. have exploited the protocol that was initially published by Lancaster et al. [49], where various brain areas self-differentiate without the constraint of added patterning factors, in order to show that the mesodermal progenitors that are naturally present develop into functional microglial cells that are integrated within the neuronal network and are capable of eliciting an immune response in cerebral organoids [92]. In another approach, where microglial progenitors and cortical organoids have been generated separately and then fused together into assembloids, microglial cells have been shown to be physiologically functional, infiltrating organoids, extending ramified processes and proving able to secrete cytokines in response to the neuronal environment [93], as well as to interact with synapses, and support neuronal maturation and bursting activity [94].

Thus, although these new approaches considerably increase the complexity of the 3D structures that are generated in vitro, they offer the possibility of modeling cellular interactions and thereby of addressing the issues that are associated with postnatal brain maturation.

### 5.3. Cell Death at the Core of the Organoid

Another limitation in modeling human cortex development with organoids is the maximum size that they can reach overtime (4–5 mm), and the necrotic core that gradually appears due to the lack of oxygen and nutrients reaching the center of the structure [95]. This considerably limits the thickness of the viable neuroepithelial structures that develop at the surface of the organoid, constituting a technical barrier to a reliable analysis of the cortical structures, as it reduces the development of both the progenitor zones (especially the oSVZ) and the neuronal layers whose boundaries are not as distinct as in the fetal brain. This also precludes long-term analyses of the later stages which are key steps in the understanding of progressive microcephaly. To overcome this diffusion limit and improve organoid survival overtime, attempts are being made to generate vascularized organoids or to develop slicing methods where the developing tissue is permanently in contact with nutrients and oxygen. Using a co-culture system in which EBs that were obtained from hiPSCs and endothelial cells that were derived from umbilical veins were induced by dual SMAD inhibition, Shi et al. obtained vascularized organoids recapitulating human cortical development [96]. The vascularized organoids promoted the development of bRG cells, resulting in a faster growth with thicker neuroepithelia and a reduced amount of cell death and hypoxia as compared to non-vascularized organoids. The laminar structure of the neocortex was also well reproduced, and the organoids could be kept in culture up to 200 days with good representation of the pyramidal neurons, interneurons, glial and microglial cells [96]. The importance of vascularization for the metabolic state of the organoid and the development of its architecture had already been demonstrated by Fred Gage’s group, who transplanted organoids into the cortex of immunodeficient mice [97]. The organoids that were transplanted in vivo were nourished by the host vascular system and showed a necrosis-free organization with mature neurons that were capable of electrophysiological activities. Alternative approaches to maintain the long-term viability of cortical organoids have recently been illustrated using organotypic slice culture. Giandomenico et al. applied this technique in maintaining slices at the air–liquid interface (ALI-COs), thereby improving the oxygen supply while retaining contact with the culture media [98,99]. The resulting organoids could be maintained up to a year while growing and maturing with minimal cell death. ALI-COs further developed mature neuronal morphology and impressive subcortical axon tracts, projecting throughout the whole culture and capable of axon guidance behaviors and functional outputs. In another study, Qian et al. obtained mature organoids with well-separated upper and deep cortical layers by making 500 μm slices that were maintained in permanent agitation and re-sliced every 4 weeks (SNOs) [37]. Interior hypoxia and cell death were considerably reduced and the resulting structures were larger and healthier, mimicking the architecture of late-stage human cortical development. These studies indicate that improved protocols can overcome the necrotic core problem and model later stages of cortical development, such as layer expansion and maturation of the cortical plate; the gradual apparition of astrocytes, oligodendrocytes and interneurons, and their interactions with excitatory neurons.

### 5.4. Cellular Stress Pathways in Organoids

Quantitative statistical analyses of gene expression during cortical maturation suggest that neuronal and glial identities, as well as gene regulatory mechanisms, methylation and transcriptional maturation signatures, are mostly recapitulated in human organoids. Using scRNA-seq to compare gene co-expression patterns between primary brain samples and cortical organoids, Pollen et al. found that although organoid cell composition can vary across experiments, developmental programs driving neuronal differentiation, radial glia maturation, cortical arealization, and metabolic states are robustly conserved in mature organoids up to 105 days [100]. scRNA-seq that was performed on ALI-COs at various stages up to one year also indicated the generation of the full repertoire of cortical neuron types and maturity markers [98]. Using functional genomic assays on long-term cultures, Gordon et al. further illustrated substantial parallels between cortical organoids and in vivo development, suggesting that mature organoids can acquire a number of postnatal features [101]. In parallel with these promising data, Bhaduri et al. recently reported that cortical organoids undergo the activation of several cellular stresses that could interfere with cell identity and cell maturation [102]. From a large set of transcriptomic analyses that were used to compare primary and organoid cortical cells, they came to the conclusion that many aspects of neuronal maturation are impaired in organoids, including heterogeneous areal identities and the activation of glycolysis and ER stress pathways that are likely to impair cell-type specification. In particular, the glycolysis gene *PGK1*, and the ER stress genes *ARCN1* and *GORASP2*, were found to be upregulated in several organoid datasets, irrespective of the differentiation protocol that was used. A significant alteration in the expression of these genes could make the modeling of microcephaly using organoids uncertain, given that variants of *PGK1* and *ARCN1* [103,104] have precisely been identified in microcephalic patients, and especially as primary microcephaly has been shown to be associated with prolonged ER stress deregulation [105]. Aware of this issue, Gordon et al. also analyzed the expression of these genes in their cortical organoids every 100 days until an advanced maturation of 600 days; however, this was without confirming any particular evolution overtime, suggesting an absence of progressive stress in their model [101]. This indicates that the regulation of stress and metabolic pathways are technically highly dependent on experimental procedures and that these challenges can be overcome by improved organoid methodologies.

## 6. Conclusions

Brain organoid technology, particularly the cortical organoid model, has developed considerably in less than 10 years and has demonstrated its rich potential for modelling microcephaly and for providing valuable information for understanding the mechanisms underlying human brain development. It is likely that the coming years will see further substantial improvements in the methodologies that are likely to homogenize differentiation and allow the routine study of very advanced stages of brain maturation. In this respect, the field of microfluidics has begun to interact with organoid technology with the aim of using biomechanical microsystems to control the fluidic environment of the organoid and thus improve its maturation [106,107]. Considering environmental parameters is all the more important, since complex interactions between genetic factors and environmental constraints probably partly explain the clinical heterogeneity that is observed between microcephalic patients, sometimes mutated in the same gene, and the significant variability in their cognitive outcomes. There is no doubt that models with improved quality, longevity and reproducibility, as well as microenvironments closely mimicking brain-specific extracellular matrix cues and allowing the control of exogenous factors, will improve our understanding of the mechanisms of microcephaly and inform new strategies for screening molecules to improve the cognitive outcome of these patients.

## Data Availability

Not applicable.

## Supplementary Materials

The following are available online at https://www.mdpi.com/article/10.3390/cells11142135/s1, Table S1: Primary Microcephalies modeled using pluripotent stem cell-derived organoids, Table S2: Secondary Microcephalies modeled using pluripotent stem cell-derived organoids.
