# Supplementary material for: Cortical Organoids to Model Microcephaly
Source: Cells. 2022 Jul 7;11(14):2135. doi: 10.3390/cells11142135 (PMC9320662; doi:10.3390/cells11142135)
Supplement: Supplementary file 1 [file cells-11-02135-s001.zip › cells-1702283-supplementary.pdf]

Table S1. Primary Microcephalies modeled using pluripotent stem cell-derived organoids

Primary Microcephaly<br><br>Acronym/<br>*Gene involved*	Organoid Model and stage	Phenotype described	Mechanism/ Molecular pathway	Year	reference
**Genetic**					
**MCPH2/**<br>*WDR62*	Dual SMAD inhibition<br>CRISPR/Cas9-induced mutations<br>4-6-week organoids	Smaller organoid size, impaired survival of NPCs and neurons, reduced NPCs proliferation, premature NPCs differentiation, increased cilia length	Delayed cilia disassembly, extended cell cycle length, premature cell cycle exit of NPCs, WDR62-CEP170-KIF2A pathway	2019	[52]
**MCPH3/**<br>*CDK5RAP2*	Unguided neural induction<br>Patient-derived hiPS cells<br>3-4-week organoids	Smaller organoid size, Decreased RG cells, increased neurons, increased of oblique and vertical oriented spindle in aRG cells	Premature neural differentiation at the expense of progenitors	2013	[15]
**MCPH5/**<br>*ASPM*	Dual SMAD inhibition<br>Patient-derived hiPS cells + RNAi knockdown organoids<br>5-12-week organoids	Smaller organoid size, Strongly disorganized neuroepithelium<br>No neuronal lamination observed	Proliferation deficiency of NPCs	2017	[48]
**Seckel type4/**<br>*CPAP*	Unguided neural induction<br>Patient-derived hiPS cells<br>2-6-week organoids	Smaller organoid size, reduced neuroepithelium, disorganized VZ with larger lumen, no difference in apoptotic cell death, increased of vertical oriented spindle in aRG cells, increased cilia length and number	Delayed cilia disassembly, extended cell cycle length leading to premature differentiation of progenitors	2016	[49]
**NEDMILG/**<br>*NARS1*	Dual SMAD inhibition<br>Patient-derived hiPS cells<br>4-13-week organoids	Smaller organoid size, disorganized neuroepithelium, thinner VZ with larger lumen, reduced number of post-mitotic neurons	Defects in RG cells proliferation	2020	[53]
**Environmental**					
**ZIKA/**<br>ZIKV infection	Dual SMAD inhibition, Spinning bioreactor<br>2-12-week Organoids from hiPS cells	Smaller organoid size, Reduced area and thickness of VZ	Increased apoptosis<br>Reduced proliferation	2016	[20]
	Unguided neural induction<br>1-8-week organoids from hES cells	Smaller organoid size, reduced NPC survival	ZIKV activates TLR3<br>TLR3 triggers apoptosis and attenuates neurogenesis	2016	[58]
	Dual SMAD inhibition<br>1-3-week organoids from hiPS cells	Reduced proliferation of aRG cell, premature differentiation	Spindle misorientation in aRG cells causing reduced proliferation, apoptosis and differentiation	2016	[59]
	Dual SMAD inhibition organoids from hiPS and hES cells	Increased apoptosis of NPCs, reduced VZ, disrupted cortical layers	N/A	2017	[57]

Table S2. Secondary Microcephalies modeled using pluripotent stem cell-derived organoids

**Secondary Microcephaly**<br><br>**Syndrome acronym/**<br>*Gene involved*	**Organoid Model and stage**	**Phenotype described**	**Mechanism/ Molecular pathway**	**Year**	**reference**
**RTT/**<br>*MECP2*	Dual SMAD inhibition + Ventral Forebrain organoids + fusion dorsal & ventral organoids<br>Patient-derived hiPS cells<br>5-12-week organoids	Marked reduction of BPs (Decreased bRG and almost no IP cells), premature neuronal differentiation, altered calcium dynamics and reduced electrophysiological responses in neurons, reduced migration capacity of interneurons	Neuronal maturation impairments, Defects in the balance between excitation and inhibition	2020	[60]
**DEE/**<br>*WWOX*	Unguided neural induction<br>CRISPR/Cas9-induced mutations<br>7-24-week organoids	Normal VZ/SVZ architecture but abnormal expression levels of cortical layer markers, increased DNA damage in aRG cells with reduced apoptosis, marked increased astrocytes, normal VGLUT1 but increased GAD67 expression, neuronal hyperexcitability	Enhanced differentiating RG cells, Wnt signaling pathway activation that would cause a disruption of neuronal specification	2021	[63]
**COH1/**<br>*VPS13B*	Dual SMAD inhibition<br>Patient-derived neurospheres<br>2-week neurospheres	Smaller neurosphere size reduced NPCs proliferation deregulation of synapse-related genes	N/A	2020	[67]
